# Supplementary figures and images for: Node properties of biomarkers within the protein–protein interaction network derived from breast cancer-associated genes
Source: PLoS One. 2026 May 6;21(5):e0347551. doi: 10.1371/journal.pone.0347551 (PMC13148703; doi:10.1371/journal.pone.0347551)

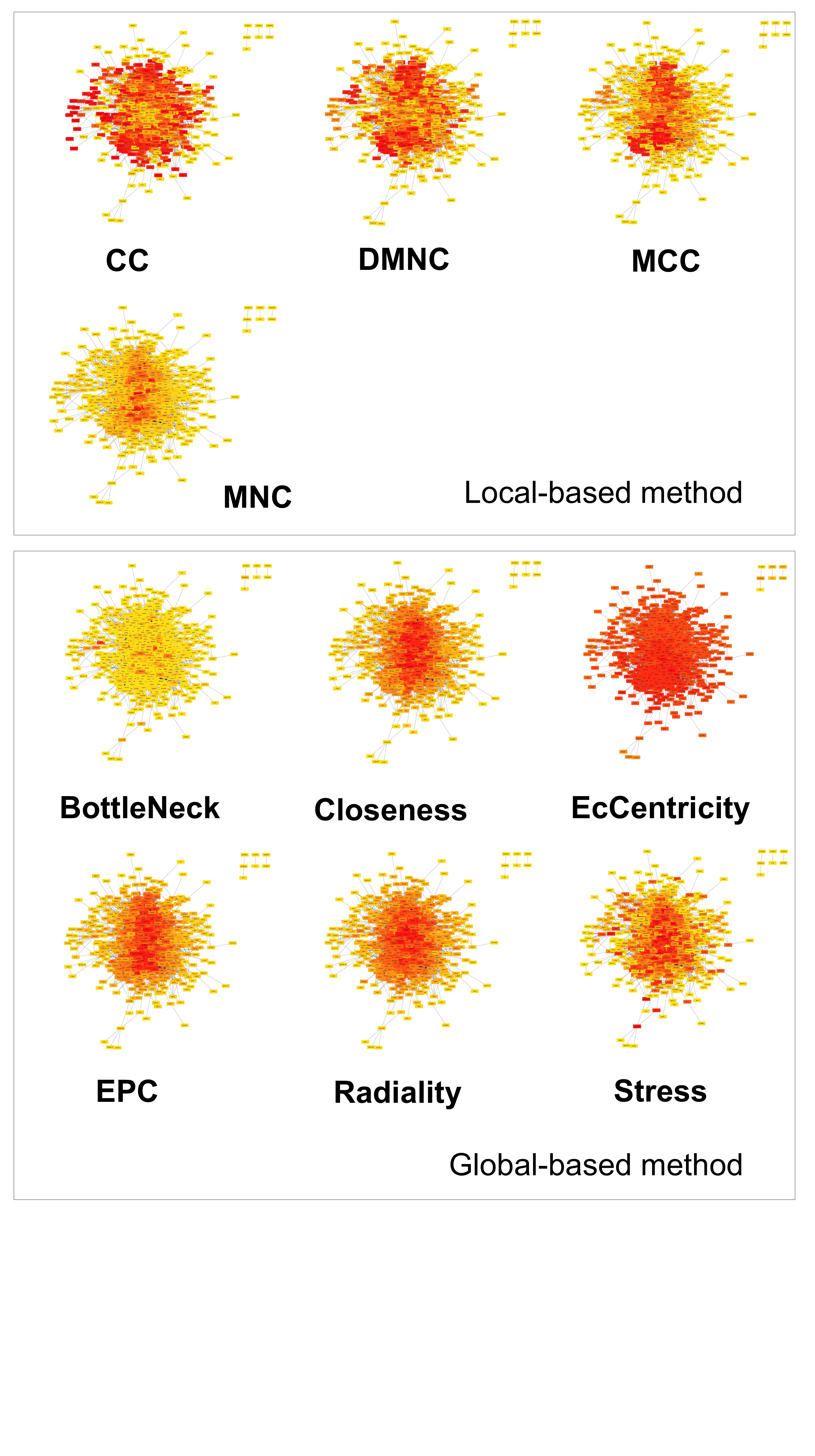

Supplement: S1 Fig — This figure displays the results for ten types of centrality analyses (excluding degree and betweenness). The analyses were performed on a PPI network of 920 nodes, which were derived from 1,000 query genes and registered in the STRING database. Medium confidence edges (minimum required interaction score = 0.4) were used. Deeper red node colors indicate higher centrality scores, and nodes with a degree of zero are not shown. CC: Clustering Coefficient; DMNC: Density of Maximum Neighborhood Component; EPC: Edge Percolated Component; MCC: Maximal Clique Centrality; MNC: Maximum Neighborhood Component; PPI: protein–protein interaction. (TIF) [file pone.0347551.s001.tif]
