# Supplementary material for: Node properties of biomarkers within the protein–protein interaction network derived from breast cancer-associated genes
Source: PLoS One. 2026 May 6;21(5):e0347551. doi: 10.1371/journal.pone.0347551 (PMC13148703; doi:10.1371/journal.pone.0347551)
Supplement: Attachment — Submitted filename: review_plosone.pdf [file pone.0347551.s012.pdf]

This study offers a clear and impactful analysis of breast cancer biomarkers within protein-protein interaction networks. By applying 12 centrality measures to networks built from 2,000 top-ranked BC-associated genes, the authors showed that maximum clique centrality (MCC) most effectively identifies known biomarkers, capturing about 36% and revealing a unique ranking pattern. High-MCC genes are enriched in cell-cycle and fibroblast growth factor receptor pathways, are evolutionarily conserved, and tend to form protein complexes. These results highlight that effective biomarkers often reside in large, conserved network cliques, providing a powerful framework for discovering new candidates. The authors' work is both methodologically rigorous and biologically insightful, offering valuable contributions to cancer research.

Comments

1. In lines 217-218, the authors state that *"The optimal condition was the network constructed from 1,500 query genes (1,362 STRING nodes) with medium confidence interactions, which showed the highest inclusion rate."* As shown in Table 1 (line 146), the 2,000-gene network contained more biomarkers (72 vs 64) and more nodes yet performed less optimally. That is an interesting finding. Could the authors provide some insight into why a larger network did not yield better results in this case?
2. In lines 125-130, the authors state that "*Pairwise comparisons were performed between H. sapiens and five other eukaryotic species*" and that "*an evolutionary conservation score was assigned to each biomarker, with 0.2 points awarded for the presence of an ortholog in each species, for a maximum possible score of 1.0.*" This is an interesting approach to measuring evolutionary conservation. Could the authors kindly share the rationale for assigning equal weight to each species? Given that D. rerio is evolutionarily much closer to H. sapiens than S. cerevisiae or A. thaliana, would a weighted scoring approach that accounts for phylogenetic distance have been considered?
3. Given that MCC inherently favors densely interconnected clique-forming genes, the authors noted in lines 343-344 that DMNC was the second-best performing metric. Since the four signaling candidates (FGF1, FGFR2, PTPN11, and ERBB3), as described in lines 331-335 and 394-398, exhibit lower connectivity to known biomarkers and appear to operate through parallel signaling pathways, could the authors kindly discuss the potential for a dual-metric ensemble approach? Specifically, would a rank aggregation of MCC and DMNC provide a more robust capture of parallel signaling pathways that might not reach the strict clique-density threshold of MCC alone, thereby strengthening the overall biomarker discovery framework proposed in this study?
4. In lines 432-433, the authors conclude that " *The analytical approach using the MCC score, as employed in this study, is a valuable method for identifying potential cancer biomarkers.*" This is a significant and promising conclusion that extends beyond breast cancer. Notably, the GO enrichment analysis in Table 3 revealed "lung development" as the 9th most significant term ($P = 3.7\times10^{-13}$), alongside breast cancer-related processes. Could the authors kindly discuss whether this suggests the MCC-based framework is capturing a universal pan-cancer core of clique-forming genes, and whether this approach would yield comparable biomarker detection rates in other cancer types?

Minor Comments

1. Could the authors please check Lines 331, and, 387 and consider changing “HMMR1” to “HMMR” to stay consistent with the official gene symbol used in the rest of the paper?"

Despite these comments (which could be incorporated in the text, if the authors feel for that) I think this work is original and important and will animate further research in this direction.
