# Supplementary material for: Node properties of biomarkers within the protein–protein interaction network derived from breast cancer-associated genes
Source: PLoS One. 2026 May 6;21(5):e0347551. doi: 10.1371/journal.pone.0347551 (PMC13148703; doi:10.1371/journal.pone.0347551)
Supplement: Attachment — Submitted filename: Editor_doc.docx [file pone.0347551.s013.docx]

Dear Editor,

I have reviewed the manuscript titled Node properties of breast cancer biomarkers within a protein–protein interaction network. Below are my notes:

- This study presents an elegant and methodologically rigorous approach to breast cancer biomarker discovery, leveraging the power of PPI network analysis to uncover biologically meaningful candidates. The proposed MCC-based framework is a timely and valuable contribution to the field.
- It is nicely written, with clear and concise language throughout.
- The structure is well organized, which enhances readability and comprehension.
- The flow of the paper is smooth and logical, making it easy for readers to follow the arguments and conclusions presented.

Overall, I find the manuscript to be of high quality and believe it would make a valuable contribution to your journal.

Best regards,

Suchana Chakravarty
